# Unusual Long-Chain *N*-Acyl Homoserine Lactone Production by and Presence of Quorum Quenching Activity in Bacterial Isolates from Diseased Tilapia Fish

**DOI:** 10.1371/journal.pone.0044034

**Published:** 2012-08-29

**Authors:** Chien-Yi Chang, Chong-Lek Koh, Choon-Kook Sam, Xin-Yue Chan, Wai Fong Yin, Kok Gan Chan

**Affiliations:** 1 School of Molecular Medical Sciences, Centre for Biomolecular Science, University of Nottingham, Nottingham, United Kingdom; 2 Natural Sciences and Science Education AG, National Institute of Education, Nanyang Technological University, Singapore, Singapore; 3 Division of Genetics and Molecular Biology, Institute of Biological Sciences, Faculty of Science, University of Malaya, Malaysia; The Scripps Research Institute and Sorrento Therapeutics, Inc., United States of America

## Abstract

Growth-dependent cell-cell communication termed quorum sensing is a key regulatory system in bacteria for controlling gene expression including virulence factors. In this study five potential bacterial pathogens including *Bacillus* sp. W2.2, *Klebsiella* sp. W4.2, *Pseudomonas* sp. W3 and W3.1 and *Serratia* sp. W2.3 were isolated from diseased Tilapia fish in Malaysia, supplied by the leading global fish supplier. Proteolytic activity assays confirmed that with the exception of *Klebsiella* sp. W4.2, all isolates showed distinct proteolytic activity. Furthermore *Bacillus* sp. W2.2 and *Pseudomonas* sp. strains W3 and W3.1 also displayed haemolytic activity. By using high resolution liquid chromatography mass spectrometry, we revealed the presence of unusually long-chain *N*-(3-oxohexadecanoyl)-homoserine lactone (3-oxo-C16-HSL) from *Pseudomonas* sp. W3.1 and *N*-dodecanoyl-homoserine lactone (C12-HSL) from *Serratia* sp. W2.3, respectively. Interestingly, *Pseudomonas* sp. W3.1 also produced a wide range of *Pseudomonas* quinolone signalling (PQS) molecules. *Pseudomonas* sp. W3 did not show any quorum sensing properties but possessed quorum quenching activity that inactivated AHLs. This study is the first documentation that shows unusual long-chain AHLs production in *Serratia* sp. and *Pseudomonas* sp. isolated from diseased fish and the latter also produce a wide range of PQS molecules.

## Introduction

Tilapia (*Oreochromis niloticus* as majority) is an important commercial fish in freshwater aquaculture around the world. The global tilapia production reached 2.5 million tonnes worth 3.7 billion US dollars in 2009 (www.fao.org). Malaysia is one of the major tilapia production countries. In 2009, a tilapia epidemic of unknown etiology broke out in Malaysia and according to the Malaysian agriculture authority, it resulted in an estimated death of about 50% of the tilapia stock in Malaysia. Preliminary investigations suggested that the causal agent of the epidemic was of bacterial origin (K. G. Chan, unpublished) which led to this work.

Many bacterial virulence factors are regulated in a population density-dependent manner known as quorum sensing (QS) which involves the synthesis of small, often diffusible signal molecules. As the bacterial population grows, the QS signaling molecules in the extracellular environment accumulates. Once the concentration of the signaling molecules reaches the threshold level, they diffuse back into the bacterial cell, binds to theirs cognate receptor and trigger changes in gene expression in response to population density [1], [2]. *N*-acyl homoserine lactone (AHL) is one of the well characterized QS signal molecules. Different AHLs possess a homoserine lactone ring with an attached fatty acyl side chain of 4 to 18 carbons [1], [3]. The acyl side chain may be saturated or unsaturated with a hydroxy or oxo group on carbon 3. AHL production is widespread among *Proteobacteria* present in a wide variety of environmental niches [4].

Interrupting QS signaling has been suggested as a promising approach to reduce the expression of QS-regulated virulence factors. This is known as quorum quenching (QQ) [3], [4]. Enzymatic inactivation of AHLs can be achieved by either opening the lactone ring moiety using an *N*-acylhomoserine lactonase (AHLase) or detaching the *N*-acyl side chain from the lactone ring via an AHL acylase [5], [6], [7]. Besides degradation, AHLs can be modified by oxidation [8], [9]. The AHL degrading enzymes are widely distributed among both prokaryotes and eukaryotes. It is not surprising that the AHLase exists in bacteria in order to reduce QS “noise” from their neighbors and prevent unnecessary gene expressions. In eukaryotes AHLase exists in order to disrupt communications among pathogens during infection, thereby acting as a defensive mechanism [9].

Here, we describe five bacterial strains isolated from tilapia which died as a result of the 2009 epidemic outbreak in Malaysia. Two of the bacteria strains produced long acyl side chain AHLs that has not been documented previously.

## Results and Discussion

### Bacterial Isolates

**Figure 1:**
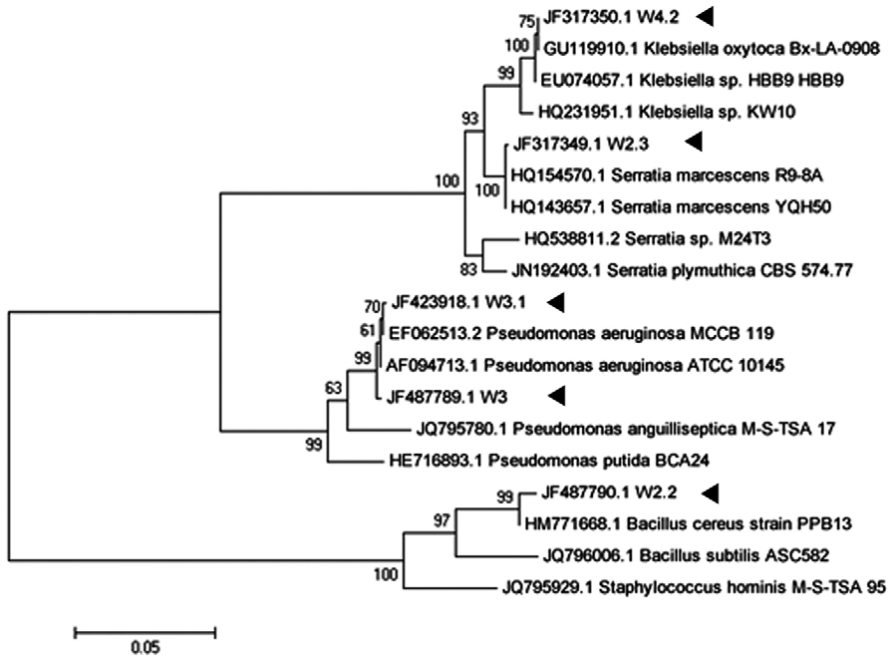
Phylogenetic analysis of the 16S rDNA sequences of isolated bacterial strains. The tree is based on maximum-likelihood (Tamura-Nei model) analysis. Bootstrap values (expressed as percentages of 1,000 replications) are shown at branch points. Solid triangles indicate the positions of isolated bacteria in phylogenetic analysis. Bar represents evolutionary distances as 0.05 changes per nucleotide position. W4.2: *Klebsiella* sp.; W2.3: *Serratia* sp.; W3 and W3.1: *Pseudomonas* sp.; and W2.2: *Bacillus* sp.

Five bacterial isolates from diseased fish were identified based on their 16S rDNA sequences, which were PCR amplified, sequenced, aligned and matched against similar sequences in NCBI GenBank. Phylogenetic results suggested these isolates belonged to two major phyla, namely Firmicutes (one isolate) and Proteobacteria (four isolates). The latter were further subgrouped into three genera: *Klebsiella*, *Serratia* and *Pseudomonas*. The single isolate in phylum Firmicutes was *Bacillus* (Figure 1). The 16S rDNA sequences of isolates *Serratia* sp. W2.3, *Pseudomonas* sp. W3, *Pseudomonas* sp. W3.1, *Bacillus* sp. W2.2 and *Klebsiella* sp. W4.2 have been deposited in GenBank under the following accession numbers: JF317349.1, JF487789.1, JF423918.1, JF487790.1 and JF317350.1, respectively.

*Streptococcus agalactiae* is a major bacterial pathogen that poses the most severe threat of morbidity and mortality to freshwater fish species around the world [10]. However, none of the five bacterial isolates was identified to be *S. agalactiae*. These results highlight the rising potential of bacterial pathogens other than *S. agalactiae* that might exist in the Malaysian aquaculture environment. However more work needs to be done to prove this speculation.

### Virulence Factor

**Figure 2:**
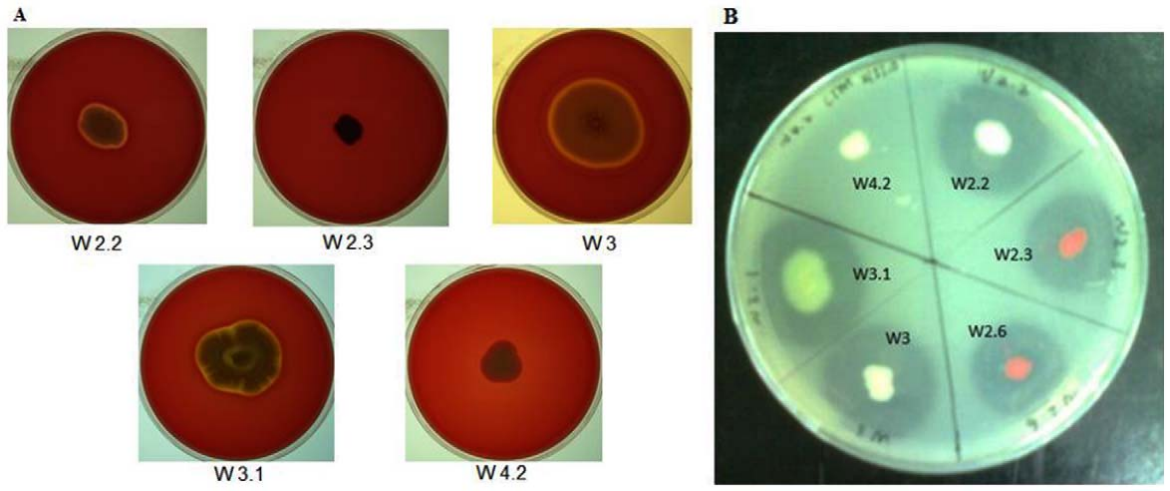
Extracellular enzyme activity of isolated strains. The overnight cultures of various strains were spotted on (A) 5% v/v sheep blood agar for haemolytic activity and (B) 1.5% v/v skim milk agar for proteolytic activity. A transparent halo zone around the colony was considered to indicate enzyme production. W2.6 showed high similarity with W2.3 in their 16S rDNA sequences (data not shown), hence only isolate W2.3 is selected for further analysis.

Isolates W2.2, W3 and W3.1 were positive for haemolytic activity (Figure 2A). Furthermore, except W4.2, all other bacterial isolates possessed proteolytic activity (Figure 2B). The ability to produce haemolysin and extracellular proteases suggested that the cultivated bacterial isolates possess nutrients acquisition from the host cells [11].

### AHL Production by Isolated Bacteria

**Figure 3:**
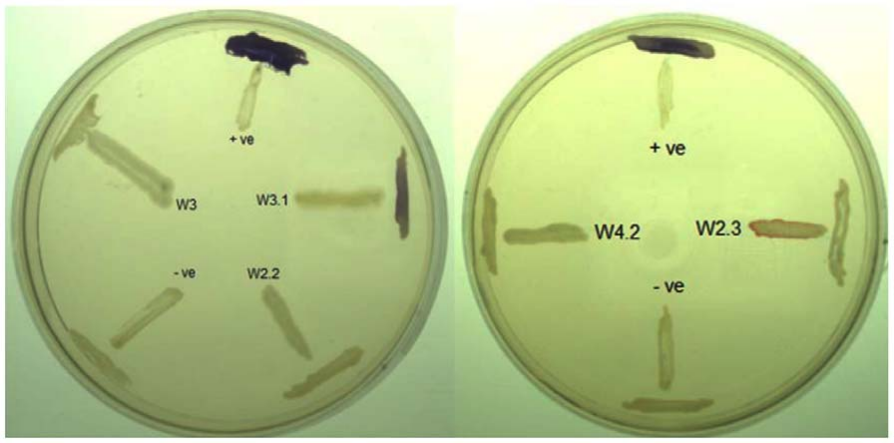
Screening of AHL production using *Chromobacterium violaceum* CV026 as biosensor. The test strains were cross-streaked against CV026. The positive control (+ve) strain is *E. carotovora* GS101 and the negative control (−ve) strain is *E. carotovora* PNP22 (AHL synthase deficient mutant). Only W3.1 isolate showed positive response.

In many Gram-negative bacterial pathogens, QS is the key regulatory system that controls the expression of virulence factors [2], [4], [12]. *Pseudomonas* sp. W3.1 was the only isolate capable of inducing violacein production in *Chromobacterium violaceum* CV026 (Figure 3), a versatile and easy-to-use reporter that responds to AHLs with 4–8 carbon acyl side chains, implying AHL production (Figure 3) [13]. It is not surprising that no AHL production was detected from *Bacillus* sp. W2.2 owing to a lack of AHL QS in Gram-positive bacteria. Interestingly, no AHLs were detected by CV026 when cross-streaked with *Serratia* sp. W2.3, *Pseudomonas* sp. W3 and *Klebsiella* sp. W4.2, although close relatives of these strains have been reported to produce AHLs detectable by CV026 [13]–[16]. This could be due to the lack of a functional *luxI* homologue gene for production of AHLs or the amount of AHLs produced by these strain was below the detection threshold of CV026. This latter hypothesis would indeed concur with the findings of Zhu *et al*., [17], [18] who reported that a small group of their clinical *Pseudomonas* spp. produce only trace amounts of AHLs not detectable by CV026. This low expression of AHLs has been found to be due to a deficient *las*I homologue gene [17], [18]. The growth medium may also affect the production of AHLs [13], [14].

### Characterization of AHLs by Mass Spectrometry

**Figure 4:**
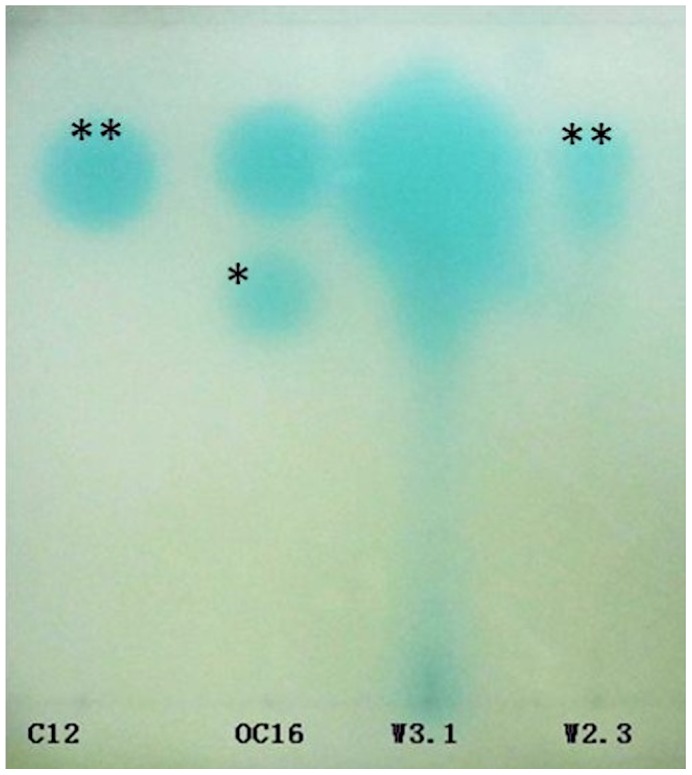
Thin layer chromatography analysis of AHLs. AHL standards from Sigma (C12-HSL (**) and 3-oxo-C16-HSL (*)) and 10 µl of W3.1 and W2.3 extract were spotted on normal phase TLC plate. The TLC plate was chromatographed with dichloromethane and methanol as mobile phases. The locations of AHLs were revealed by overlay of *Agrobacterium tumefaciens* NTL4 pZLR4 as evident by blue pigmentation. Based on the R*_f_* values, W2.3 produced C12-HSL.

**Figure 5:**
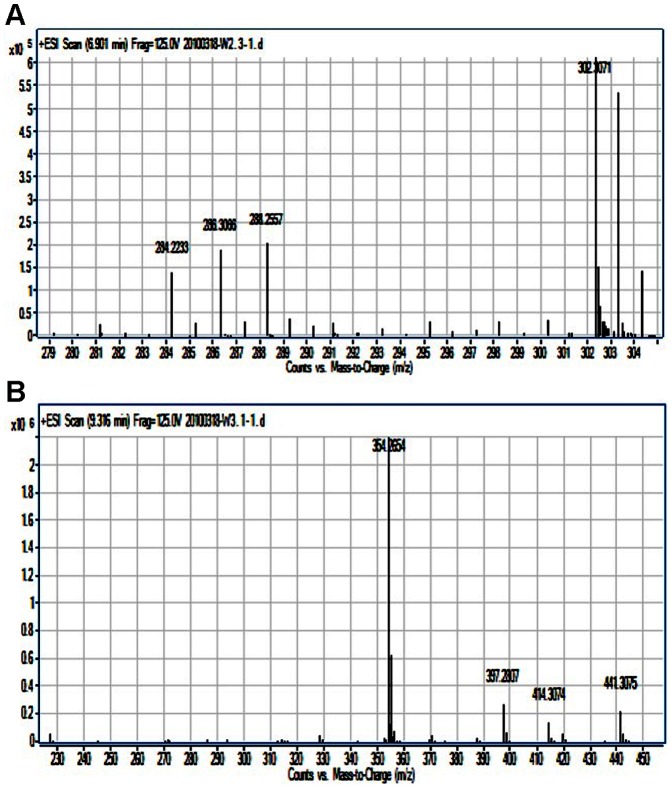
High resolution mass spectrometry analysis of AHLs. (A) ESI-MS spectrum of C12-HSL (*m/z*:284.2233; 6.90 min) extracted from *Serratia* sp. W2.3; (B) ESI-MS spectrum of 3-oxo-C16-HSL (*m/z*:354.2654; 9.32 min) extracted from *Pseudomonas* sp. W3.1.

Spent supernatants of *Serratia* sp. W2.3 and *Pseudomonas* sp. W3.1 cultures were assayed for AHLs using thin layer chromatography (TLC) and high resolution liquid chromatography mass spectrometry (LCMS) as described in this work. Based on the retention factor (R*_f_*) of the TLC analysis, the extract of *Serratia* sp. W2.3 showed the presence of C12-HSL (Figure 4). The result of high resolution LCMS confirmed that C12-HSL (*m/z*:284.2233; 6.90 min) was present in the spent supernatant of *Serratia* sp. W2.3 (Figure 5A).

In *S. marcescens* SS-1, the well-studied SpnIR QS system regulates flagellum-independent population surface migration and synthesis of biosurfactant, prodigiosin, and nuclease [15]. Also, SpnI synthesizes at least four short-chain AHLs including *N*-3-(oxohexanoyl)-homoserine lactone (3-oxo-C6-HSL), *N*-hexanoyl-homoserine lactone (C6-HSL), *N*-heptanoyl-homoserine lactone (C7-HSL), and *N*-octanoyl-homoserine lactone (C8-HSL) [15]. Contrary to many reports that *S. marcescens* produces only short chain AHLs, in this study, *Serratia* sp. W2.3 did not produce short chain AHLs as it failed to activate *C. violaceum* CV026 biosensor but it produced an unusual long chain C12-HSL, which was identified by TLC and LCMS. These results implied that AHL synthase of *Serratia* sp. W2.3 is capable of synthesizing long chain AHLs, a phenomenon that has not been reported previously.

Similarly, a long chain 3-oxo-C16-HSL (*m/z*:354.2654; 9.32 min) (Figure 5B) has also been detected by high resolution LCMS from *Pseudomonas* sp. W3.1. Ortori and colleagues [13] have profiled the AHLs production from *P. aeruginosa* PAO1 laboratory strain by using LC-MS/MS. To further verify that 3-oxo-C16-HSL is the only AHL produced, extract of *P. aeruginosa* W3.1 was analyzed by using this published method developed by Ortori and co-workers [13]. To our surprise, a range of AHLs and quinolones were detected namely: *N*-butanoyl-L-homoserine lactone (C4-AHL) (*m/z*:172); *N*-(3-oxododecanoyl)-L-homoserine lactone (3-oxo-C12-AHL) (*m/z*:298); 2-heptyl-4-hydroxyquinolone (C7-AQ) (HHQ) (*m/z*:244); 2-nonyl-4-hydroxyquinolone (C9-AQ) (NHQ) (*m/z*:272); 2-heptyl-4-hydroxyquinoline *N*-oxide (C7-N-oxide) (HQNO) (*m/z*:260); 2-nonyl-4-hydroxyquinoline *N*-oxide (C9-N-oxide) (NQNO) (*m/z*:288); 2-heptyl-3-hydroxy-4(1H)-quinolone (C7-PQS) (*m/z*:260) and 2-hydroxy-2-nonyl-4(1H)-quinolone (C9-PQS) (*m/z*:288) (Table 1). To the best of our knowledge, this is the first documentation of such a wide range of AHL and quinolone molecules being detected in environmental *Pseudomonas* strain.

**Table 1:** Mass spectrometry identification of different quorum sensing signaling molecules from bacterial isolates spent supernatants.

	C4-HSL	3-oxo-C12-HSL	C7-AQ	C9-AQ	C7-N-oxide	C9-N-oxide	C7-PQS	C9-PQS
Sample Name	172.1/102.1	298.1/102.1	244.1/159.1	272.1/159.1	260.1/159.1	288.1/159.1	260.1/175.1	288.1/175.1
Synthetic standards	1.06E+07	1.50E+07	4.57E+05	1.77E+06	1.84E+07	2.45E+05	5.83E+05	5.75E+05
W2.2	0.00E+00	0.00E+00	0.00E+00	0.00E+00	0.00E+00	0.00E+00	0.00E+00	0.00E+00
W2.3	0.00E+00	0.00E+00	0.00E+00	0.00E+00	0.00E+00	0.00E+00	0.00E+00	0.00E+00
W3	0.00E+00	0.00E+00	0.00E+00	0.00E+00	0.00E+00	0.00E+00	0.00E+00	0.00E+00
W3.1	1.49E+07	2.42E+05	2.20E+05	1.75E+06	1.74E+07	2.26E+05	1.32E+06	5.29E+05
W4.2	0.00E+00	0.00E+00	0.00E+00	0.00E+00	0.00E+00	0.00E+00	0.00E+00	0.00E+00

Intriguingly, in sharp contrast to *Pseudomonas* sp. W3.1, the spent supernatant of *Pseudomonas* sp. W3.1 did not contain any detectable AHL and quinolone molecules. It is well established that bacteria use QS as a means to synchronize cooperative behaviours at the population level [9]. However, evolutionary theory predicts that individuals who communicate and cooperate can be exploited [19]. Using the opportunistic pathogen *P. aeruginosa*, Diggle and co-workers show that social cheaters can derive the advantage and benefit of QS while avoiding the cost of producing the QS signalling molecules or performing the cooperative behaviour that is coordinated by QS, and therefore act as riders in the population [19]. The presence of haemolysin and extracellular proteases while avoiding the production of AHL and quinolone molecules by *Pseudomonas* sp. W3 may provide the evidence to Diggle’s discovery of social cheater in bacteria population. Our finding suggest that *Pseudomonas* sp. W3 could produce haemolysin and extracellular proteases without QS circuit clearly suggest that they are able to exploit their kin population on proteases activity which is usually QS-mediated [19].

Furthermore, a study suggests that the 3-oxo-C14-HSL is the longest acyl side chain AHLs recorded for the AHLs produced by marine *Pseudomonas* sp. [20]. To date, no AHLs with acyl side chain longer than 14 carbons have been reported. This is the first study to identify an unusual 16-carbon side chain AHL (3-oxo-C16-HSL) produced by a *Pseudomonas* strain, suggesting that the isolate W3.1 has a broad AHL production profile.

*Rhodobacter capsulatus* and *Sinorhizobium meliloti* have been reported as exhibiting C16-HSL production which is involved in the regulation of gene transfer and nodule initiation, respectively, in these species [21]–[23]. A recent report show the LasR-based bioluminescent biosensor successfully detected the long-chain AHLs including four diverse AHLs of the C16-HSL family expressed by the *avsI* gene of *Agrobacterium vitis* [24]. This suggested the *P. aeruginosa* could sense these long-chain AHLs and respond to this signaling in certain situations. This, however, requires further work to be carried out to identify the role of 3-oxo-C16-HSL in virulence regulation in *Pseudomonas* sp. W3.1.

### QQ Activity of Isolated Bacteria

**Figure 6:**
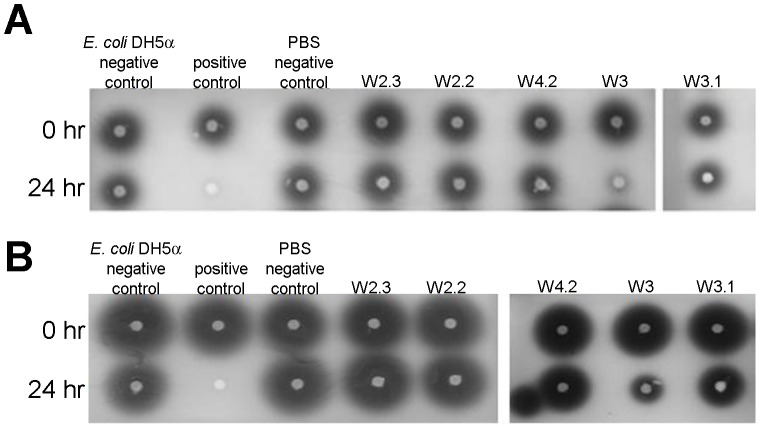
Degradation of AHLs by isolated strains. Residual C4-HSL (A) and C6-HSL (B) were detected by *C. violaceum* CV026. Decreased or loss of violacein indicated AHL inactivation after incubation for 24 h. *B. cereus* known for carrying a lactonase was used as positive control. *E. coli* DH5α and PBS served as negative controls.

There is no detectable AHLs from *Pseudomonas* sp. W3 and *Klebsiella* sp. W4.2 extractions that could be identified by either CV026 biosensor or high resolution LCMS. A Previous study suggested the tropical marine *Pseudomonas* sp. and topical montane forest soil *P. frederiksbergensis* exhibit both QS and QQ abilities [20], [25]. Hence, it is not surprising that *Pseudomonas* sp. W3 which was suspected to be a social cheater was able to efficiently degraded C4-HSL and C6-HSL (Figure 6). Also, *Bacillus* spp. are well-known for harboring AHL lactonase and thus inactivating QS signals by hydrolysing the lactone ring of AHLs [8], [16], [26].

*B. cereus, which* is known for producing strong lactonase, was used as positive control, similarly *Escherichia coli* DH5α and PBS was used as a negative control [27]. Only *Pseudomonas* sp. W3 appeared to degrade C4-HSL and C6-HSL by revealing decreased or loss of violacein pigment after 24 h (Figure 6) but not the *Bacillus* sp. *P. aeruginosa* PAO1 has been determined as encoding an AHL acylase gene, the product of which targets 3-oxo-C12-HSL but not C4-HSL [28]. This is the first study showing the degradation of C4-HSL by *Pseudomonas* sp. W3 without any detectable AHLs production. However it has been reported that one tropical marine *Pseudomonas* strain produces C12-HSL and 3-oxo-C14-HSL and also degraded C6-HSL, 3-oxo-C6-HSL and 3-oxo-C8-HSL [20]. Although in the phylogenetic analysis, *Pseudomonas* sp. W3 and W3.1 showed a close phylogenetic relationship and they both have haemolytic and proteolytic activities, their QS and QQ characterizations suggested that extracellular enzyme activities may not be regulated by QS or QQ networking. Similar findings indicate the complexity of QS even in the same species of *Pseudomonas* [17].

A recent study identified *Klebsiella* spp. that exhibit a broad spectrum of AHL-degrading activities *via* lactonolysis associated with the rhizosphere of ginger (*Zingiber officinale)* growing in the Malaysian rainforest involving in the polymicrobial community involving both QS and QQ bacteria [29]. However the *Klebsiella* sp. W4.2 in this study shows no QS and QQ characteristics. Also this isolate lacked haemolytic and proteolytic activities.

## Materials and Methods

### Isolation and Characterization of Virulence of Bacteria from Tilapia

Fish samples were collected, and transported on ice (not frozen) to laboratory for immediate examination in order to identify the course of the epidemic outbreak of tilapia fish in 2009. After wiping the fish’s surface with 70% v/v ethanol to remove the surface contaminations, fish samples were dissected using sterile scalpels. Sterile swabs were wiped through the inner muscle 1 cm below the skin and immersed in Phosphate Buffer Saline (PBS) (100 mM, pH 6.5). The solution was then serially diluted and spread on lysogeny broth (LB) agar plates before incubating overnight at 28°C.

Virulence factors of the isolates were determined by subjecting the isolates to Columbia 5% v/v sheep blood agar (BD, USA) and 1.5% v/v skim milk agar (0.05% w/v yeast extract, 0.1% w/v tryptone, 1% w/v NaCl and 1.5% v/v skim milk, solidified with 1.5% w/v Bacto agar). The blood agar was used to examine the haemolytic activity of the isolates while the skim milk agar was used to examine the proteolytic activity.

### 16S rDNA Identification and Phylogenetic Analysis

In order to better identify the bacterial isolates, their genomic DNA were extracted with QIAamp Mini Kit (Qiagen, USA) and 16S rDNA PCR were performed using universal bacterial 16S rDNA primers mentioned previously [27]. The 1.5 kb PCR-amplified products were cloned into pGEMT plasmid (Promega, USA) in *E. coli* DH5α and sequenced for phylogenetic analysis. The sequence alignment for the phylogenetic tree was constructed and evaluated with MEGA 5 software based on maximum-likelihood (Tamura-Nei model) analysis [30].

**Table 2:** Summary of phenotypic properties of isolated bacterial strains from infected tilapia.

Strain	16S Identity	GenBank Accession Number	Hemolytic Activity	Proteolytic Activity	QS Signaling Molecules Detectedby LCMS (*m/z* ratio)	QQ Activity
W3.1	*Pseudomonas* sp.	JF423018.1	+	+	C4-HSL (*m/z*:172)	−
					3-oxo-C12-HSL (*m/z*:298)	
					3-oxo-C16-HSL (*m/z*:354.26)	
					C7-AQ (*m/z*:244)	
					C9-AQ (*m/z*:272)	
					C7-N-oxide (*m/z*:260)	
					C9-N-oxide (*m/z*:288)	
					C7-PQS (*m/z*:260)	
					C9-PQS (*m/z*:288)	
W2.3	*Serratia* sp.	JF317349.1	−	+	C12-HSL (*m/z*:284.22)	−
W2.2	*Bacillus* sp.	JF487790.1	+	+	N.D^a^	−
W3	*Pseudomonas* sp.	JF487789.1	+	+	N.D^a^	+
W4.2	*Klebsiella* sp.	JF317350.1	−	−	N.D^a^	−

N.D^a^ Not Detectable.

+ Positive result.

− Negative result.

### AHL Detection

To investigate whether these isolates exhibited an AHL-based QS regulation system, bacterial cultures were cross-streaked with the AHL biosensor CV026 which produced violacein, a violet pigment, in the presence of exogenous short chain AHLs ranging from 4–8 carbons and mainly non-substituted in acyl side chain [31]. For AHLs detection, *Erwinia carotovora* GS101 and *E. carotovora* PNP22 served as positive and negative controls, respectively [32]. Analysis of AHLs by TLC method was performed as previously described [16].

### LCMS Analysis of AHLs from Isolated Bacteria

LCMS has been proven to be a suitable method for the detection of QS signals [13]. In order to compensate the detection limit of biosensor CV026, high resolution LCMS analysis provides unequivocal confirmation of AHL identification. A single colony of bacteria was cultured overnight in 5 ml of LB medium buffered with 50 mM 3-(*N*-morpholino) propanesulfonic acid (MOPS). Briefly, 1 ml of the culture was transferred into 200 ml of fresh LB-MOPS and incubated at 28°C for 18 h, 150 rpm. The spent supernatant was then extracted three times with an equal volume of acidified ethyl acetate (0.1% v/v acetate acid). The extract was evaporated to dryness and stored at −20°C.

Prior to LCMS analysis, the dried extract was dissolved in 150 µl of acetonitrile. Then, 100 µl of the dissolved extract was used for LCMS analysis. Liquid chromatography was carried out on Agilent RRLC 1200 system and the high resolution mass spectrometry was carried out on Agilent 6500 Q-TOF MS/MS system. The column used for analysis was Agilent ZORBAK Rapid Resolution HT column. The mobile phases used were acetonitrile and ultrapure water. The analysis was conducted at 45°C with the flow rate of 0.4 mL/min.

The detection of variation of QS signals produced by isolated bacteria was carried out by analyzing 2 ml of the spent supernatants by LCMS using the published method as illustrated by Ortori and colleagues [13] which simultaneously detect both AHL and quinolone molecules.

### QQ Activity

The five bacterial isolates were subjected to a QQ inactivation assay as described previously in order to investigate the QQ activity of each isolate [27]. Bacteria were washed and resuspended in 1 ml of PBS (100 mM, pH 6.5) with synthetic C4-HSL or C6-HSL (Sigma-Aldrich, USA) to a final concentration of 0.5 µg/µl. The mixtures were incubated for 0 and 24 h with 220 rpm shaking at 28°C and then heated at 95°C for 3 min to inactivate the reaction [33]. Aliquots (10 µl) from each sample was spotted on the CV026 lawn and incubated at room temperature for 24 h. Decreased or loss of violacein pigmentation on the CV026 lawn indicated QQ activity.

### Final Remarks and Conclusions

Rather than the common short-chain AHLs, here we report the major QS molecules produced by *Serratia* sp. W2.3 and *Pseudomonas* sp. W3.1 were C12-HSL and 3-oxo-C16-HSL, respectively, as identified by high resolution LCMS (Table 2). Our data also show the production of a wide range of AHLs and *Pseudomonas* quinolone signaling molecules by *Pseudomonas* sp.W3.1. *Pseudomonas* sp.W3 which did not produce any QS molecules may play a role as a social cheater in the environment and may be a rider that exploits QS system in natural *Pseudomonas* population.
